# The evidence synthesis and meta-analysis in R conference (ESMARConf): levelling the playing field of conference accessibility and equitability

**DOI:** 10.1186/s13643-022-01985-6

**Published:** 2022-06-03

**Authors:** Neal R. Haddaway, Alexandra Bannach-Brown, Matthew J. Grainger, W. Kyle Hamilton, Emily A. Hennessy, Ciara Keenan, Chris C. Pritchard, Jana Stojanova

**Affiliations:** 1 Leibniz-Centre for Agricultural Landscape Research (ZALF), Eberswalder Str. 84, 15374 Müncheberg, Germany; 2 Africa Centre for Evidence, University of Johannesburg, Johannesburg, South Africa; 3 Berlin Institute of Health at Charité–Universitätsmedizin Berlin, QUEST Center, Charitéplatz 1, 10117 Berlin, Germany; 4 Norwegian Institute for Nature Research, Postboks 5685 Torgarden, 7485 Trondheim, Norway; 5 Psychological Sciences, University of California Merced, Merced, CA USA; 6 Health Sciences Research Institute, University of California Merced, California, USA; 7 Recovery Research Institute, Massachusetts General Hospital, 151 Merrimac Street, Boston, MA USA; 8 School of Psychology, Queen’s University, Belfast, UK; 9 Institute of Health and Allied Professions, Nottingham Trent University, Nottingham, UK; 10 Department of Department of Clinical Pharmacology & Toxicology, St Vincent’s Hospital Sydney, Darlinghurst, Australia

**Keywords:** Online conference, Equity, Pay-it-forwards, Volunteer, Systematic review, Synthesis, Community of Practice, Evidence synthesis technology

## Abstract

Rigorous evidence is vital in all disciplines to ensure efficient, appropriate, and fit-for-purpose decision-making with minimised risk of unintended harm. To date, however, disciplines have been slow to share evidence synthesis frameworks, best practices, and tools amongst one another. Recent progress in collaborative digital and programmatic frameworks, such as the free and Open Source software R, have significantly expanded the opportunities for development of free-to-use, incrementally improvable, community driven tools to support evidence synthesis (e.g. EviAtlas, robvis, PRISMA2020 flow diagrams and metadat). Despite this, evidence synthesis (and meta-analysis) practitioners and methodologists who make use of R remain relatively disconnected from one another. Here, we report on a new virtual conference for evidence synthesis and meta-analysis in the R programming environment (ESMARConf) that aims to connect these communities. By designing an entirely free and online conference from scratch, we have been able to focus efforts on maximising accessibility and equity—making these core missions for our new community of practice. As a community of practice, ESMARConf builds on the success and groundwork of the broader R community and systematic review coordinating bodies (e.g. Cochrane), but fills an important niche. ESMARConf aims to maximise accessibility and equity of participants across regions, contexts, and social backgrounds, forging a level playing field in a digital, connected, and online future of evidence synthesis. We believe that everyone should have the same access to participation and involvement, and we believe ESMARConf provides a vital opportunity to push for equitability across disciplines, regions, and personal situations.

## ESMARConf as an active community of practice working on free and open source evidence synthesis methods and tools

The recent pandemic forced many research community activities and conferences online. Here, we report on a new online conference for evidence synthesis and meta-analysis in the R programming environment (ESMARConf), which focuses on accessibility and equity as core missions. ESMARConf is an event series geared towards a novel community of practice of tool developers and users working with evidence synthesis and meta-analysis in R. We believe this conference has the power to curate a vital community of practice across disciplines, raising awareness, fostering capacity sharing, and developing free and Open Source, efficient and rigorous needs-based tools to support evidence synthesis. ESMARConf builds on the success and groundwork of the broader R community and systematic review coordinating bodies (e.g. Cochrane), but fills an important niche; catering to a variety of audience types including developers, users and learners. Here, we introduce ESMARConf and its core values, calling for the evidence synthesis and R communities to fully embrace both the conference and this style of event in an effort to maximise accessibility and equity.

## The need for rigorous evidence synthesis methodology

Rigorous evidence is vital across disciplines for efficient, appropriate, and fit-for-purpose decision-making with minimised risk of unintended harm, and to make the best use of available resources [1]. Rigorous evidence syntheses (e.g. systematic reviews) are the most robust means of summarising bodies of evidence in a way that minimises bias and maximises comprehensiveness, accuracy, repeatability, and reliability [2–4].

To date, however, disciplines have been slow to share evidence synthesis frameworks, best practices, and tools amongst one another. Instead, evidence synthesis paradigms are borrowed but largely reinvented for new disciplines wishing to learn from other fields. This exceptionalism and limited collaboration undoubtedly results in inefficiencies and unnecessary teething problems [5, 6]. However, some efforts have been made to support interdisciplinarity in evidence synthesis: for example, in 2015, the Global Evidence Synthesis Initiative (GESI) brought together a network of centres working on evidence synthesis and knowledge translation across fields in low- and middle-income countries.

## The benefit of free and open source software environments

The importance of communities of practice has also been recognised for some time in existing interdisciplinary contexts. Developments in collaborative digital and programmatic frameworks, such as the free and Open Source software R, have significantly expanded the opportunities for development of free-to-use, incrementally improvable, community-driven tools to support evidence synthesis. Examples of such R-based evidence synthesis tools include: EviAtlas for visualising cartographic evidence maps [7]; robvis for visualising risk-of-bias assessments [8]; PRISMA2020 for visualising PRISMA-compliant flow diagrams [9]; metadat for accessing meta-analytic datasets for training and testing purposes [10]. By being Open Source, these tools ensure the evidence synthesis processes are computationally reproducible [11–14].

Although the community of R tool (i.e. package) developers is active and strong across disciplines, evidence synthesis (and meta-analysis) practitioners and methodologists who make use of R remain relatively disconnected from one another, often also existing outside and potentially unaware of systematic review coordinating bodies, such as Cochrane, the Campbell Collaboration, and the Collaboration for Environmental Evidence. Furthermore, the R community of *tool developers* is not well connected with *users* of evidence synthesis tools. This means that many needed tools may go undeveloped, and tools that could have a broad applicability may not end up being as widely used because end users are not aware of their existence or because certain use cases and needs may not be met.

## On the need for an evidence synthesis technology community of practice

Clearly, then, there is a need to develop a community of practice of evidence synthesis tool developers and users. Such an active and well-connected, interdisciplinary community would facilitate the production of fit-for-purpose, needs-driven tools that conformed to rigorous standards in evidence synthesis best practice. A community would also facilitate capacity sharing and awareness raising across the use of evidence synthesis tools and methods. Furthermore, as some have argued (e.g. [15]), this may also lead to a more creative ecosystem that develops synergistically to meet needs and to maximise efficient use of resources. In short, a more reliable, efficient, and flexible evidence ecosystem.

## The development of ESMARConf

In 2020, active members of the Evidence Synthesis Hackathon (https://www.eshackathon.org/), an organisation that aims to produce Open Source evidence synthesis tools, initiated planning of the first Evidence Synthesis and Meta-Analysis in R Conference (ESMARConf; https://esmarconf.github.io/), held on 21st and 22nd January 2021. The event consisted of 39 presentations of 7 min, 10 panel discussions, and 4 workshops. During the 4 days of the conference, the conference received 514 registrations, with 650 unique viewers during the conference and > 3500 video views.

In February 2022, our second event, ESMARConf2022 took place, consisting of 28 presentations, 3 panel discussions, and 6 workshops. The event received 843 registrations, and the content has been viewed by 1388 unique viewers, corresponding to 4945 views (as of May 1st 2022).

**Fig. 1:**
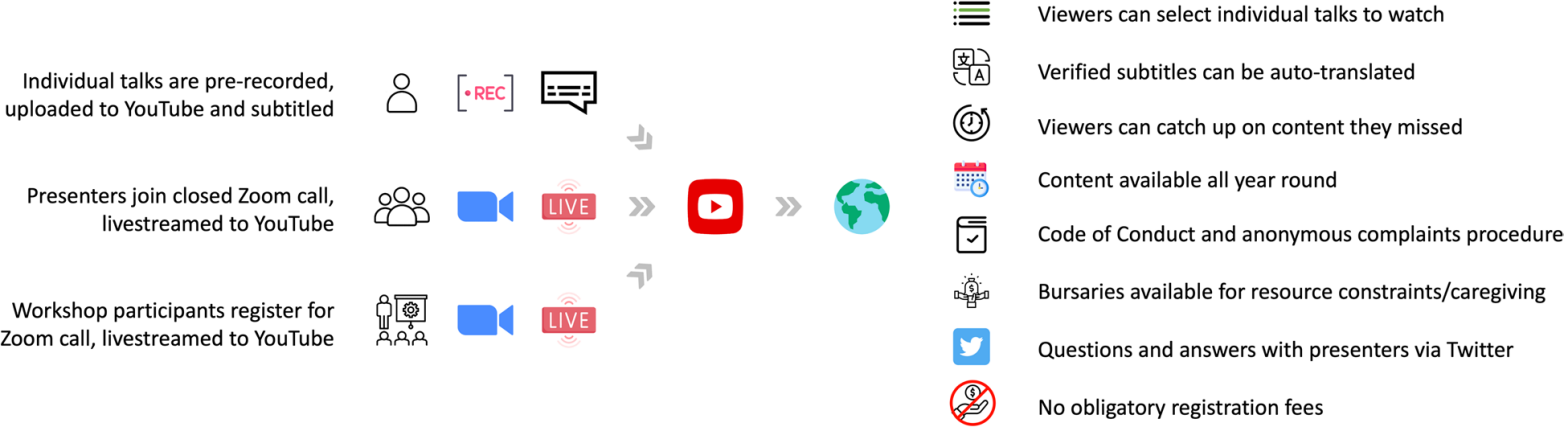
Schematic showing the organisation of ESMARConf as an online, permanently available conference

ESMARConf is a purely online conference, designed to be as accessible and equitable as possible (see Fig. 1), whilst delivering state-of-the-art and impactful content to build and share capacity, work collaboratively, and develop needs-based tools.

**Fig. 2:**
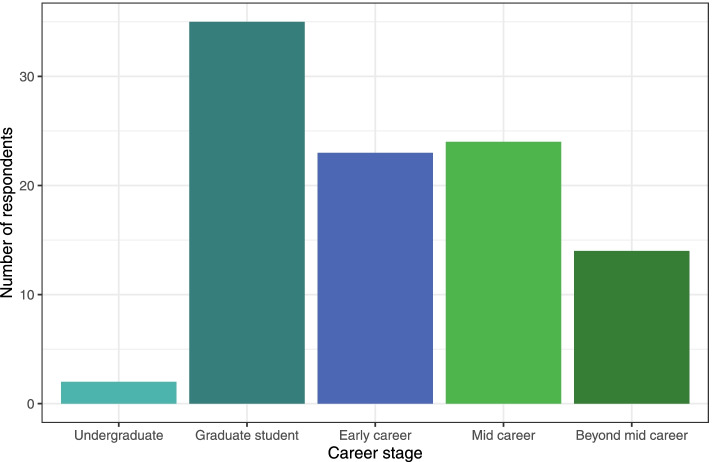
Distribution of ESMARConf2022 evaluation respondents by career stage. Data available here: 10.5281/zenodo.6397517

Importantly, we aim to combine presentations of existing tools and frameworks in R with capacity building and training both around the use of R in evidence synthesis and rigorous evidence synthesis methodology itself. By leveraging R as an open and widely used platform for statistics and data visualisation, we have the opportunity to connect a large and disciplinarily diverse audience of graduate students and earlier stage researchers with best practices in rigorous evidence synthesis. Indeed, our post-event evaluations demonstrate the importance of these career stages, but also demonstrate continued engagement from more senior researchers (Fig. 2).

We developed a mission for ESMARConf that builds on the principles and values of R both as a coding platform and a community of practice, and the communities leading and promoting rigorous systematic reviews (Cochrane, the Campbell Collaboration, and the Collaboration for Environmental Evidence).

**Fig. 3:**
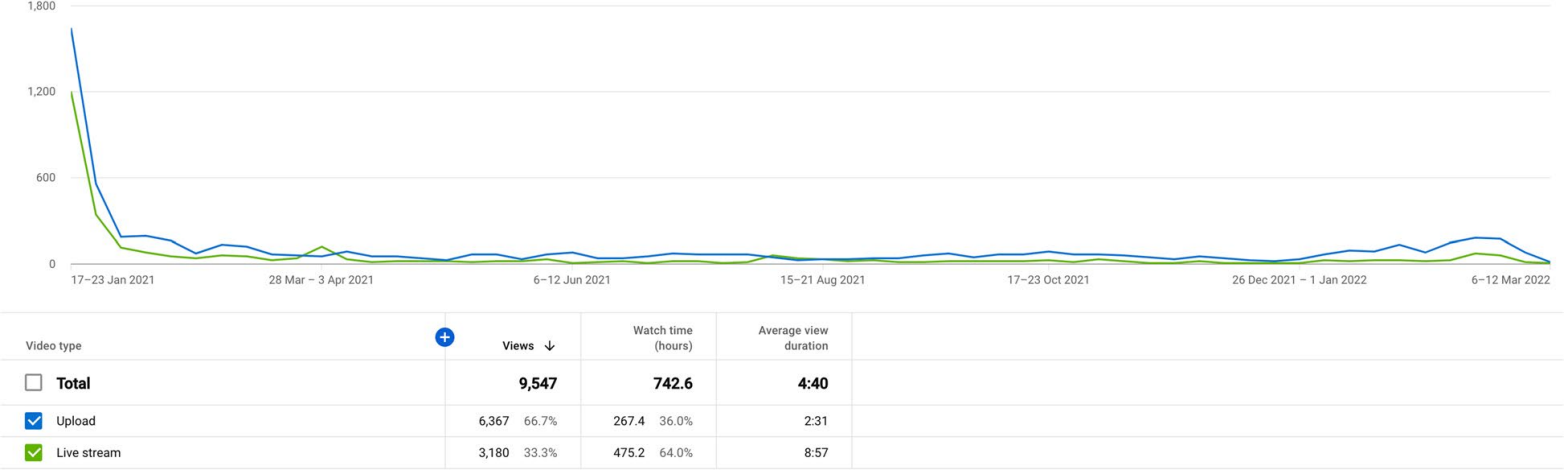
Weekly views of the ESMARConf2021 videos separated by video type (live stream in blue, pre-recorded individual talk upload in green). Screenshot from YouTube analytics. Data available here: 10.5281/zenodo.6397517

**Fig. 4:**
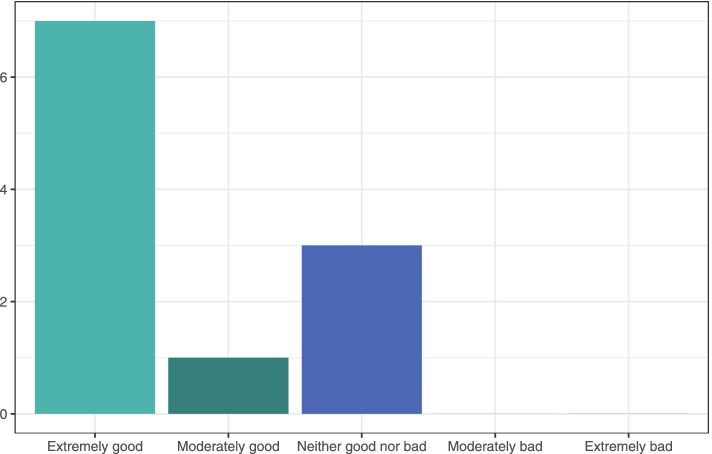
Responses to the question “Taking everything into account, how good/bad was it that you had to pre-record your talk?” from 11 presenter responses in the post-event evaluation for ESMARConf2022. Data available here: 10.5281/zenodo.6397517.

**Fig. 5:**
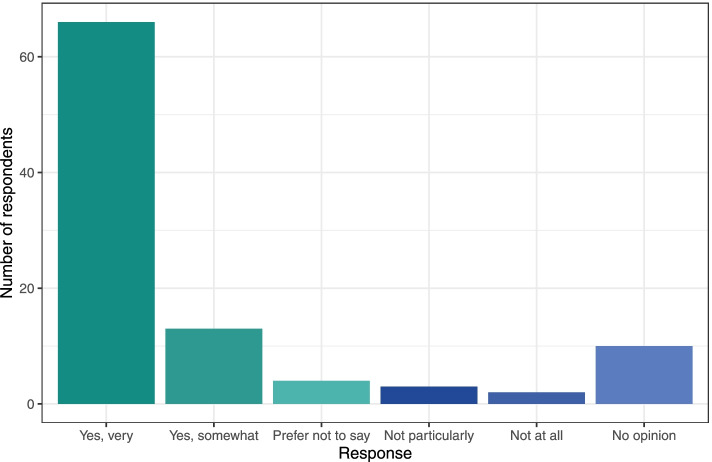
Responses to the question “Do you feel that you were personally welcome at ESMARConf2022?” from 60 participant responses in the post-event evaluation for ESMARConf2022. Green, purple and light blue segments correspond to 1 response (1.7%) each. Data available here: 10.5281/zenodo.6397517

**Fig. 6:**
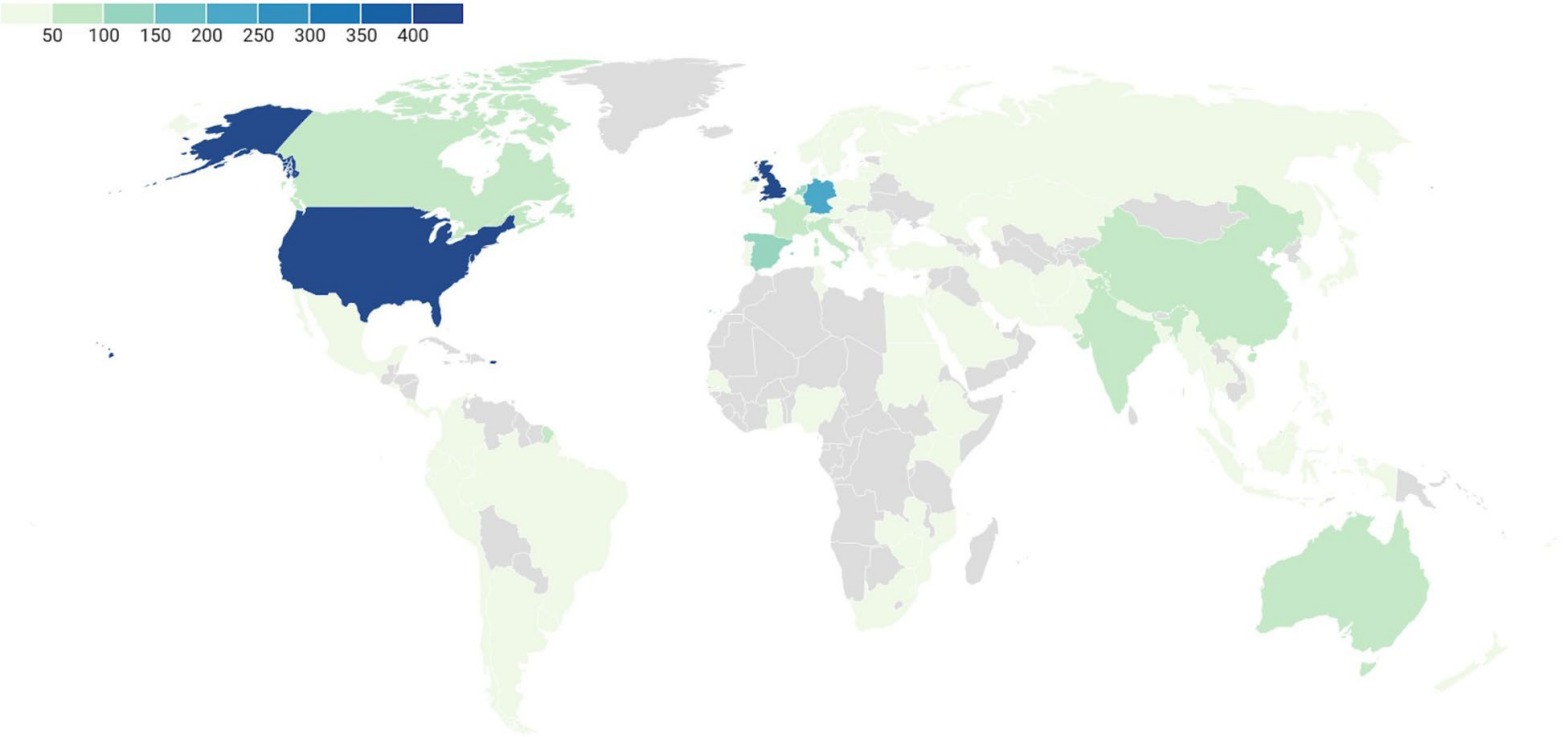
Choropleth displaying the number of participants (identified from analytics for the https://ESMARConf.github.io website) from across 86 countries between 01/01/22 and 09/03/22. Interactive version available at: https://www.datawrapper.de/_/5my3A. Produced using https://www.datawrapper.de/. Data available here: 10.5281/zenodo.6397517

We aim to make ESMARConf as accessible and equitable as possible through the following core values and initiatives:

- *Conference fees should not be a barrier to participation*. High conference fees are a huge financial barrier to those who most need access to information and networking. We believe that online events should not be costed at the same rate as traditional, in-person conferences. We believe that running online events using free and low-cost software (such as Google Forms, YouTube and Zoom) is vital for enabling low/no registration fees and facilitating equitable participation. We now have the experience of running two large, global conferences entirely for free for participants. We aim to ensure ESMARConf is free forever, but there are costs to ensuring these events are accessible (e.g., time to organise and coordinate events, translation/subtitling services), and so we welcome voluntary donations and registrations where participants’ budgets allow. We are also striving to develop Open Source automated pipelines for ESMARConf to minimise burden on conference organisers, and we welcome others who may wish to use them for the same purposes (see the *ESMARConf_updater* repository; https://github.com/ESHackathon/esmarconf_updater).

- *Online conferences remove barriers to physical attendance*. By holding the conference online and recording all live content, participants can digest the content at a time that works for them. Around 67% of the more than 9500 views of the ESMARConf2021 videos on our YouTube Channel were for individual pre-recorded talks (as opposed to the live stream), and 73% of all views occurred after the end of the conference (see Fig. 3). The vast majority of engagement with the conference happened in ‘catch-up’ rather than live, highlighting the benefit of providing content for participants to view on their own time. Furthermore, by allowing presenters to pre-record their talks and play these during the livestream, we reduce the need for physical presence and allow presenters from any time zone to take part: 73% of presenters stated that providing a pre-recording of their talk was extremely or moderately good and no respondents indicated that having to pre-record their talk was bad (Fig. 4).

- All participants should be equally welcome and valued. Accessibility and equity are vital cornerstones to the event and our community of practice. We believe that people have an equal right to take part in the conference in a safe and welcoming environment. Our Code of Conduct and complaints and feedback procedures (https://www.eshackathon.org/about/accessibility_and_codeofconduct.html) are continually evolving and central to all our events. We mention them at the start of each live session and clearly link to them on our website. Some 83% of participants responding to an evaluation of ESMARConf2022 said that they felt personally welcome (see Fig. 5).

- *Access should not be restricted by the ability to hear*. Along with attempting to ensure participants can join the conference according to their own schedule, we believe that participation should not rely on an ability to hear. To that extent, we ensure that all pre-recorded individual talks are subtitled, and that the transcripts are verified in English before the conference begins. We could not do this without the wonderful dedication of our presenters, who ensured recordings were sent in on time to allow for subtitles to be produced and checked manually, and without generous funding from Code for Science & Society to fund a team of transcribers and editors.

- *Access should not be restricted by language*. Similarly, we do not believe that language should be a barrier to taking part in ESMARConf. The verified subtitling described above allows users to automatically translate closed captions into any language provided by YouTube (currently more than 155). In future years, we hope to be able to allow presenters to present in the language of their choice in pre-recorded talks, providing subtitles that can be translated into the viewer’s preferred language.

- *Access should not be restricted by geographical location*. By running the event as an exclusively online conference, we aim to allow anyone with access to an internet connection (including mobile or satellite internet) and a charged/mains powered electronic device that can display a YouTube video to join. In particular, we aim to facilitate participation from people in traditionally underrepresented regions and countries. ESMARConf2022 was attended by participants from across 86 countries (see Fig. 6), with a substantial number of viewers (173 of 1120) coming from low- and upper-middle-income countries; something we aim to increase in the future. In general, we believe this substantially reduces barriers otherwise present for face-to-face conferences.

- *Caregiving responsibilities and resource constraints should not prevent participation*. Many conferences create barriers to participation by caregivers, including parents and those supporting people living with disabilities. Furthermore, finding time to join an online conference for multiple days is a substantial challenge for participants with caregiving responsibilities. Similarly, we understand that many potential participants face resource constraints when trying to join an online conference, particularly those in low- and middle- income countries. To support our caregiving participants and those with resource constraints, at ESMARConf2022 we provided bursaries of up to 100 USD: this funding could be put towards a wide variety of costs, including: alternative caregivers; support with meal preparation; costs of commuting to a town with strong internet connection; hiring a quiet space or a location with stable electricity; hiring a diesel generator and mobile internet; purchasing headphones, etc. In total, 26 bursaries were awarded across both caregiver and resource constraint bursary types.

## Sustainability challenges and call to action

There are clear limitations and challenges behind organising such an event. Firstly, it relies on the efforts of a voluntary organising group. From 2021 onwards, modest funding has been obtained that includes a budget line for salary costs for key organisers working to coordinate the event itself. But a broader group of volunteers to make key decisions, help advertise the conference, and facilitate sessions is still needed. Furthermore, it also relies on a sustained interest from presenters and workshop providers contributing their time for free. Funding is also needed to cover the costs of verification of subtitles, provision of bursaries, and small software costs (namely, a Zoom subscription to support live streaming to YouTube). However, these costs are not excessive for a conference that can easily be scaled up in participation without additional costs. In our evaluation of ESMARConf2022, 75% of presenters and 37% of participants supported the idea of a voluntary registration fee to support attendance by those with accessibility and resource constraints. For participants, the median voluntary registration fee people were willing to pay was 50 USD. This indicates that an annual budget of c. 15,000 USD might be possible (although the willingness-to-pay was likely higher in those choosing to respond to the evaluation). This is a promising indication that an objective to break even in the long term might be feasible, considering that our costs for ESMARConf2022 were c. 7000 USD.

ESMARConf aims to provide for three key audience groups and their objectives: (1) coders producing evidence synthesis tools wishing to collaborate; (2) current/potential R users wishing to conduct an evidence synthesis in R and learn more about the tools available; (3) non-R users with expertise/experience in evidence synthesis wishing to use free tools via non-coding user interfaces. As a result, collaboration, awareness raising, capacity building and sharing, and matching users to developers are core objectives of the conference: more generally speaking, developing a community of practice in tool use and development.

**Fig. 7:**
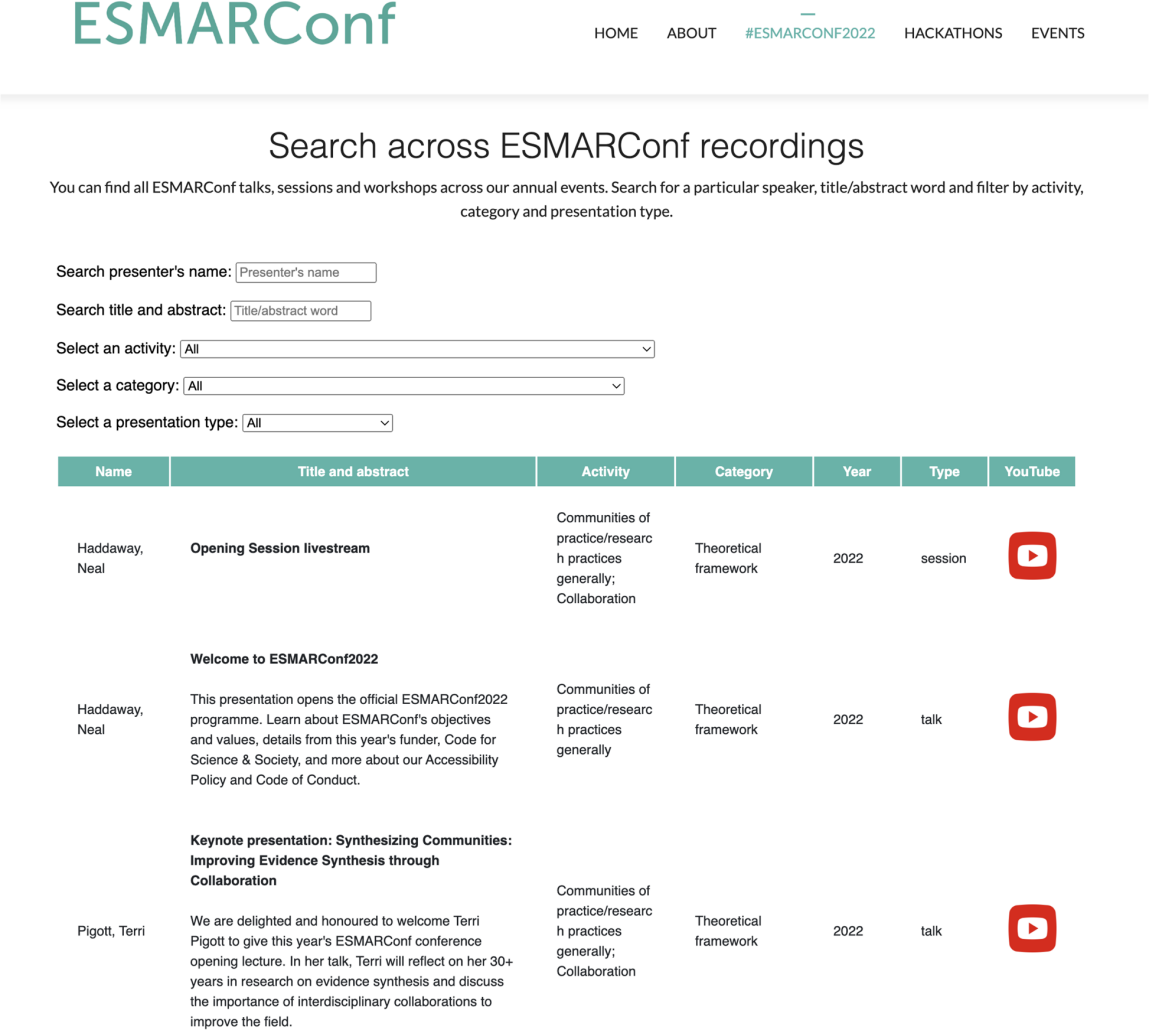
Screenshot of the searchable database of ESMARConf presentations and workshops, showing activity and category descriptors

The core values in our mission are important to ensuring engagement by all three audience groups, but we recognise several important factors that differ between groups. Firstly, we aim to support interested tool developers by matching their skills with other coders and specific projects, valuing all backgrounds and abilities, and also attempting to integrate opportunities for continued learning: all expertise are valued and everyone is learning. Secondly, we aim to make it easy to find relevant ESMARConf content for participants wishing to find tools for specific tasks. We do this by hosting an interactive database of all ESMARConf content indexed by review stage (e.g. eligibility screening or meta-analysis) and type (e.g. theoretical framework, web-based tool or R package) that can be searched and filtered (Fig. 7). Thirdly, we aim to raise awareness about the importance of rigour in evidence synthesis by providing capacity development related to methodology theory, such as the popular systematic searching workshop from ESMARConf2022 [16]. Finally, we aim to support non-coders and R novices by emphasising tools that have been developed with point-and-click user interfaces, and by encouraging developers to provide tutorials and walkthroughs of their tools: this will be a key focus for ESMARConf2023.

We call on members of our three audiences to join the evidence synthesis and meta-analysis in R community of practice by embracing ESMARConf. Whatever your background in evidence synthesis, meta-analysis or R, you can play a vital role in ensuring that free and Open Source tools are developed that are fit-for-purpose, widely used and accessible to all. By joining and supporting the community, you can help us to raise awareness, build and share capacity and improve the rigour of evidence syntheses across disciplines. Support us by registering for ESMARConf2023 next year and engaging with presenters and colleagues, by presenting your work and sharing your skills, and by helping to develop needs-driven tools to improve accessibility, efficiency, and rigour of evidence syntheses across disciplines. Where possible, donations and voluntary registration fees can ‘pay it forwards’, helping to support those with constraints to break down barriers and participate in a global community.

By supporting ESMARConf, you can help to maximise accessibility and equity across regions, contexts, and social backgrounds, helping to aim for a level playing field in a digital, connected, and online future of evidence synthesis. Welcome to ESMARConf!

## Data Availability

All data analysed are available in an open and public Zenodo dataset record: Haddaway, NR. 2022. ESMARConf2022 evaluation responses and interaction statistics [Data set]. Zenodo. 10.5281/zenodo.6397517.
